# Diagnostic confounders of chronic widespread pain: not always fibromyalgia

**DOI:** 10.1097/PR9.0000000000000598

**Published:** 2017-04-30

**Authors:** Winfried Häuser, Serge Perrot, Claudia Sommer, Yoram Shir, Mary-Ann Fitzcharles

**Affiliations:** a Department Internal Medicine I, Klinikum Saarbrücken, Saarbrücken, Germany; b Department of Psychosomatic Medicine and Psychotherapy, Technische Universität München, München, Germany; c Centre de la Douleur, Cochin Hospital, Paris Descartes University, Paris, France; d Department of Neurology, University of Würzburg, Wurzburg, Germany; e Alan Edwards Pain Management Unit, McGill University Health Centre, Montreal, QC, Canada; f Division of Rheumatology, McGill University Health Centre, Montreal, QC, Canada

**Keywords:** Fibromyalgia, Differential diagnosis

## Abstract

**Introduction:**

Chronic widespread pain (CWP) is the defining feature of fibromyalgia (FM), a worldwide prevalent condition. Chronic widespread pain is, however, not pathognomonic of FM, and other conditions may present similarly with CWP, requiring consideration of a differential diagnosis.

**Objectives:**

To conduct a literature search to identify medical conditions that may mimic FM and have highlighted features that may differentiate these various conditions from FM.

**Methods:**

A comprehensive literature search from 1990 through September 2016 was conducted to identify conditions characterized by CWP.

**Results:**

Conditions that may mimic FM may be categorized as musculoskeletal, neurological, endocrine/metabolic, psychiatric/psychological, and medication related. Characteristics pertaining to the most commonly identified confounding diagnoses within each category are discussed; clues to enable clinical differentiation from FM are presented; and steps towards a diagnostic algorithm for mimicking conditions are presented.

**Conclusion:**

Although the most likely reason for a complaint of CWP is FM, this pain complaint can be a harbinger of illness other than FM, prompting consideration of a differential diagnosis. This review should sensitize physicians to a broad spectrum of conditions that can mimic FM.

## Abstract 2

Although chronic widespread pain is the defining feature of fibromyalgia, a myriad of other conditions may present with similar pain complaint leading to misdiagnosis. Conditions that may mimic fibromyalgia may be categorized as musculoskeletal, neurological, endocrine/metabolic, psychiatric/psychological, and medication related. In this review, we examine these various conditions that should be considered in a differential diagnosis and provide direction that will help the clinician differentiate these conditions from fibromyalgia.

## 1. Introduction

Chronic widespread pain (CWP) is the cardinal symptom of fibromyalgia (FM), but may also occur as a symptom of other diseases. Failure to recognize other conditions that can masquerade as FM could adversely affect patient outcome, especially for conditions that have defined treatments other than those recommended for FM. In similar vein, some patients may be given a diagnosis of some other condition, when in fact the correct diagnosis is FM. Thus, the diagnostic confusion may be bidirectional. For this reason, it is important that physicians are vigilant in the assessment of a patient presenting with a chronic pain syndrome, and that a differential diagnosis is established before immediately assigning a diagnosis of FM. Even once a specific primary diagnosis has been made, such as an inflammatory rheumatic disease or neurological disorder, an associated FM may also be present and requires recognition and attention. With FM increasingly recognized as a cause of suffering and with prevalence rates worldwide in the order of at least 2%, physicians have become more attuned to consideration of this diagnosis, with resulting risk of over diagnosis.^98^ Indeed, overdiagnosis of “fashionable” pain syndromes is not unusual, for example when evaluating patients suspected of having complex regional pain syndrome type I.^15^

There are numerous factors that still contribute to uncertainties surrounding the diagnosis of FM, including reliance on patient report of subjective symptoms, absence of a universally accepted diagnostic gold standard, and lack of a specific biomarker. Various diagnostic and classification criteria for FM have been proposed in the last 2 decades, with considerable debate regarding the concept of FM as a unique diagnosis, or as a condition that may be concomitant with some other illness. Whereas the American College of Rheumatology (ACR) 1990 classification criteria,^97^ the 2011 (survey),^94^ and the 2016 criteria^96^ state that the diagnosis of FM is valid irrespective of any other medical condition; the preliminary ACR 2010 diagnostic criteria^95^ required the exclusion of diseases which might sufficiently explain the pain. The 2016 criteria^96^ further caution that simply satisfying FM criteria is not, ipso facto, sufficient to diagnose FM or to define the entirety of the patient's medical condition.

In the assessment of a person presenting with CWP, the total clinical context must always be addressed because other conditions, although strictly fulfilling criteria for FM, might require a completely different treatment strategy. Therefore, in line with all current guidelines for FM care, patients must be evaluated with a comprehensive medical history and examination to identify any features that could perhaps point to an alternate diagnosis.^2,27,35,56^

Diagnoses that can be confused with FM may be broadly grouped into the following categories: inflammatory rheumatic diseases, nonrheumatic musculoskeletal conditions, nonrheumatic medical conditions (endocrinology, gastroenterology, infectious diseases, and oncology), neurological conditions, mental health disorders, and medication-induced pain conditions.^43^ In this report, we will describe various diagnoses that should be considered when a patient presents with a complaint of CWP and will highlight clinical features to assist the health care professional in establishing a correct diagnosis. We will also consider a challenging situation for health care professionals, when FM can be considered as a comorbid condition, especially when associated with rheumatic or neurological diseases, or secondary to infectious diseases. This synopsis will cover most, but not necessarily all of the more common conditions presenting similar to FM.

## 2. Methods

A literature search of MEDLINE was conducted for articles published from 1990 through September 2016 using the following MeSH (Medical subject Heading) terms: “chronic widespread pain,” “fibromyalgia,” “diffuse pain,” “musculoskeletal pain,” “myalgia,” “arthralgia,” “matched with “misdiagnosis,” “differential diagnosis,” “neurological disease,” “endocrine disease,” “metabolic disease,” “rheumatic disease,” “mental health disorder,” “psychological illness,” psychiatric disease,” “medication,” “drugs,” and “drug adverse reaction.” From the references of relevant articles, we accessed additional literature relevant to the topic.

## 3. The clinical encounter

The foundation for evaluating a patient with CWP is a comprehensive history and physical examination, which may be followed by specifically directed investigations as indicated (Table 1).^2,27,35,56^ Although some tools have been developed to help primary care physicians' screen for FM, they are only a screen and should not be used to establish a diagnosis.^11,68^ As a first step, the location of pain can be assessed by means of a pain diagram and if pain is observed to be diffuse (according to the 2016 criteria), further questioning regarding associated symptoms of unrefreshed sleep and fatigue should be pursued. Positive responses in the setting of CWP would identify the condition as an FM-type syndrome. Thereafter, the clinical history must explore symptoms related to other organ systems, systemic symptoms, family history of medical and mental health illness, and also screening for psychological distress, eg, with the use of a simple instrument such as the Patient Health Questionnaire-4, an ultra-short screening tool for anxiety and depression.^53^ Attention must be given to timing of onset and evolution of symptoms, as well as alleviating or aggravating factors. The character of the pain can be variable, with some patients describing a deep gnawing pain, whereas others report a burning quality to the pain. Although an associated feature of prolonged body stiffness traditionally suggests an underlying inflammatory rheumatic process, some patients with FM can experience considerable stiffness. There are some “Yellow flags” in the history and physical examination pointing towards FM (Table 2).

**Table 1:**
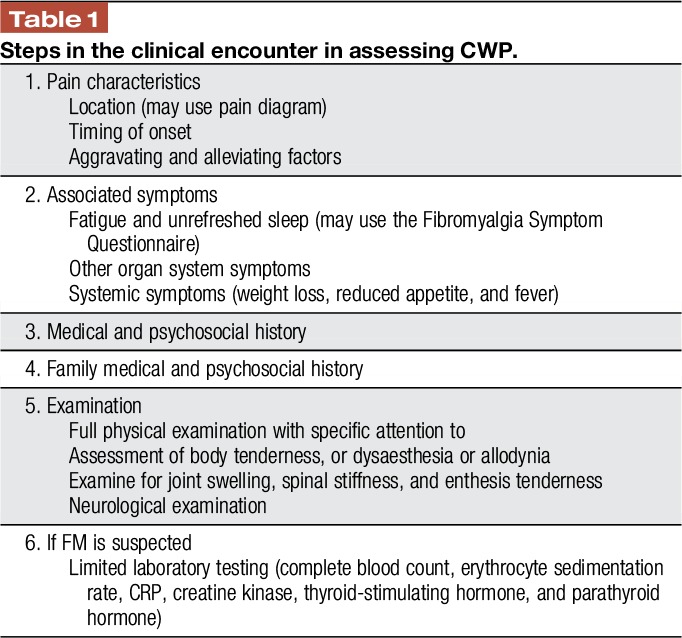
Steps in the clinical encounter in assessing CWP.

**Table 2:**
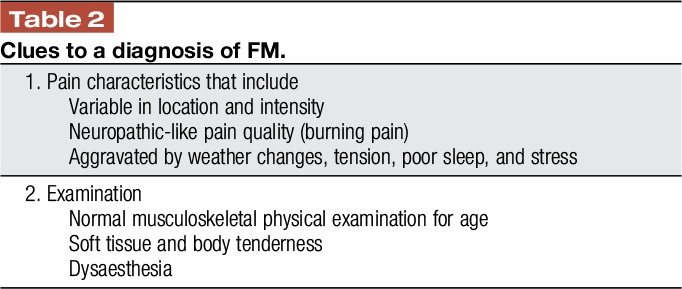
Clues to a diagnosis of FM.

Although a patient may have a preconceived idea that symptoms are due to FM or perhaps have been previously diagnosed with FM by some other health care professional, this diagnosis should not be accepted uncritically. Furthermore, a physical examination is required specifically to examine for evidence of structural joint abnormality, muscle weakness, neurological abnormality, or evidence of endocrine disease. Thereafter, additional testing may be prompted by information obtained from the clinical assessment. An effective clinical evaluation, therefore, carries important weight in the evaluation of a patient with a complaint of body pain. Only limited investigations are required if the clinical assessment points to FM (Table 1). A summary of conditions that should be considered in the differential diagnosis of a patient presenting with CWP, as well as “red flags” characteristic for each, and suggested specific testing are detailed below (Table 3).

**Table 3:**
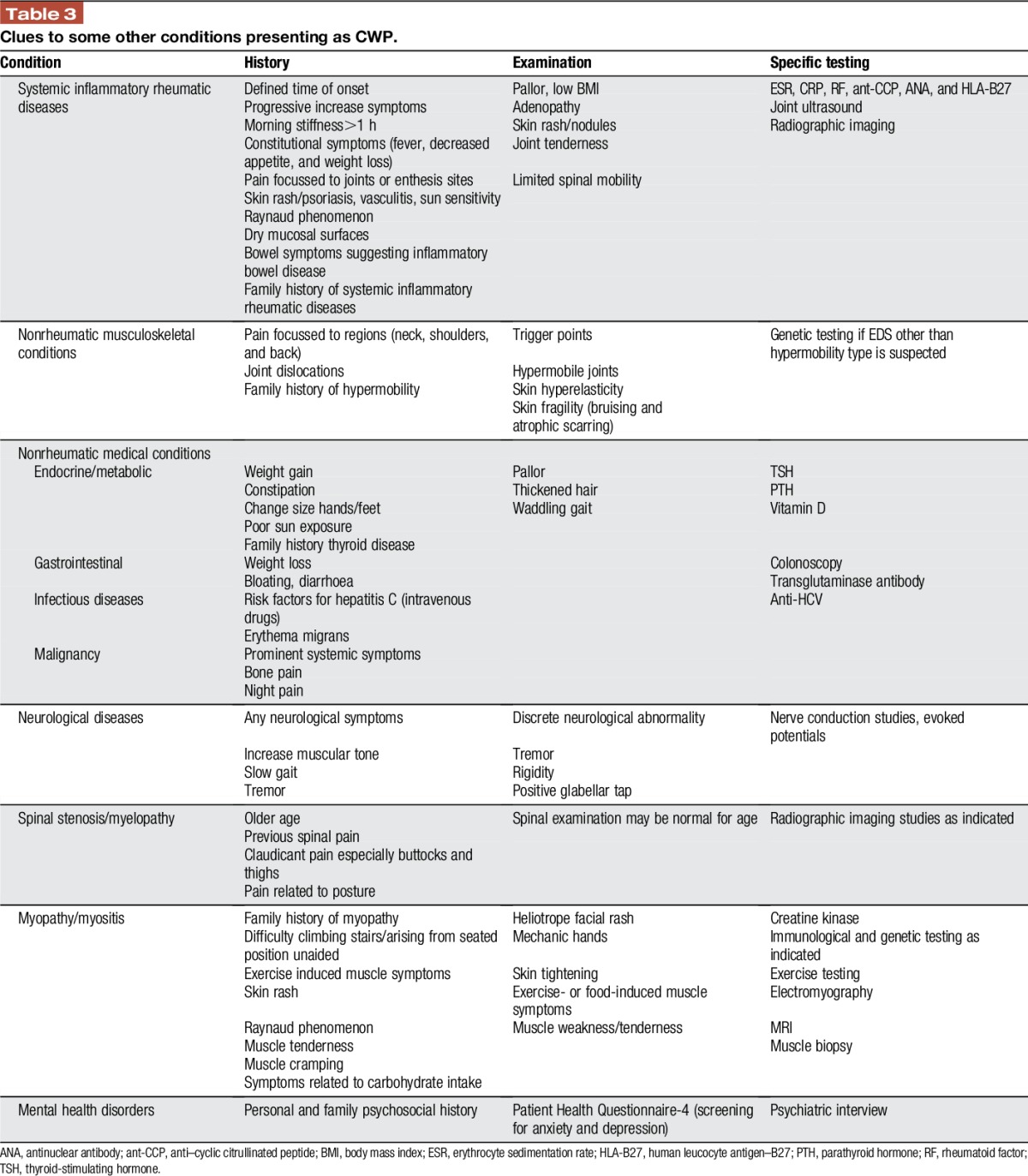
Clues to some other conditions presenting as CWP.

## 4. Systemic inflammatory rheumatic diseases

Although patients with an early stage of a systemic inflammatory rheumatic disease may have generalized body pain, it can be expected that identifiable physical or laboratory abnormalities will develop over time, but with caution that a single abnormal laboratory test is generally not sufficient evidence to diagnose an inflammatory rheumatic disease.^32,33^ Although joint swelling is the hallmark of many inflammatory rheumatic diseases, some may have less prominent joint symptoms and rather present with a diffuse pain syndrome, but often with accompanying systemic symptoms or additional clues on the history. Therefore, the early stages of conditions such as rheumatoid arthritis (RA), systemic lupus erythematosus (SLE), Sjogren syndrome, mixed connective tissue disease, scleroderma, or inflammatory spondyloarthritis (SpA) may present with an FM-like onset.^69^ In many cases, this also raises the question of comorbid FM that aggravates the quality of life and increases the burden of the disease for these conditions.^76^ It must also be remembered that a patient may previously have had FM, but then may at a later time develop another disease. The following are descriptions of some common rheumatic conditions that may initially present similar to FM. “Red flags” gathered by medical history and physical examination indicating potential inflammatory rheumatic disease are summarized in Table 3.

### 4.1. Inflammatory polyarthritis

Peripheral inflammatory arthritis (IA) is a symptom of various rheumatic conditions including RA, psoriatic arthritis, SLE, and others, and may present in the early stages as a diffuse musculoskeletal pain before the development of definite joint synovitis. Preclinical RA can cause fatigue, stiffness, and even muscle weakness in addition to pain, before the development of overt joint swelling.^60,90^ There are usually some clues on history or examination that could prompt consideration of a diagnosis alternate to FM. The nonspecific presenting symptoms will, however, evolve into definite expression of joint disease over time, which could take several months.

The red flags that may suggest IA as a possible diagnosis rather than FM include a family history of IA, morning stiffness longer than an hour, more severe constitutional symptoms such as weight loss, a progressive increase in symptom severity, pain symptoms focussed in the joints, other associated features such as skin rash, Raynaud phenomenon, sun sensitivity, dry mucosal surfaces, and prominent bowel symptoms, especially diarrhoea. Examination findings that may point towards a diagnosis of IA include tenderness more localized to the joints, especially the joints of hands and feet, skin rashes such as psoriasis, malar rash, and livedo reticularis or vasculitis changes especially at the nail fold regions, adenopathy, or salivary gland enlargement for Sjogren syndrome, and any abnormality of examination of other systems. Testing that will help identify an IA include measurement of inflammatory markers such as erythrocyte sedimentation rate, C-reactive protein (CRP), rheumatoid factor, anti–cyclic citrullinated peptide antibodies, and other immunological parameters such as antinuclear antibody as indicated. Ultrasound of joints can show synovitis and inflammation.

### 4.2. Polymyalgia rheumatica

Polymyalgia rheumatica (PMR) is a common inflammatory rheumatic disease with an incidence rate of about 50 cases per 100,000 persons aged 50 years and older in the United States, twice as common in women compared with men, and a cause of severe suffering and debility if untreated. Although classically described as pain and stiffness involving the shoulder and buttock regions, PMR may also present as a more diffuse pain, but usually with prominent stiffness.^24^ Pain that disturbs sleep and fatigue can be present, and with prolonged symptoms mood disorder may develop. In the presence of severe headache, jaw claudication or visual disturbance and temporal artery tenderness, giant cell arteritis, which may occur in up to 10% of those with PMR, should be considered.

Some clues to differentiate PMR from FM include an older age of onset; a more clearly defined time of onset over a few weeks in a person without previous severe musculoskeletal pain; prominent night pain; new onset of systemic symptoms of fatigue, weight loss and poor appetite; prolonged stiffness in the mornings; limitation of range of motion of shoulders, or mild peripheral synovitis. Elevated inflammatory markers, erythrocyte sedimentation rate or CRP, will help support a diagnosis of PMR, although these may be normal in a small proportion of patients. Various imaging techniques such as ultrasound examination of the subacromial bursa or the temporal artery or magnetic resonance imaging (MRI) are promising additional studies to demonstrate areas of inflammation.^57,82^ As corticosteroids are the treatment mainstay, a positive response to a therapeutic trial of corticosteroids will help consolidate the diagnosis. Some patients with FM may, however, report a limited response to corticosteroids, possibly as a result of steroid-induced energy boost, although the response is not as pronounced as that seen for PMR.

### 4.3. Inflammatory spondyloarthritis

The distinction between inflammatory SpA and FM is currently emerging as an important diagnostic challenge. The reason is that new criteria for a diagnosis of SpA include soft tissue rheumatism in the form of enthesitis and also allow for diagnosis of less advanced disease without prominent radiographic findings. Ankylosing spondylitis, previously remembered as occurring mostly in men and resulting in a fused or bamboo spine, is now recognized to occur in less advanced forms, equally affecting men and women, and may even be diagnosed in the absence of radiographic sacroiliitis, a previous hallmark of the disease. In fact, according to newer criteria, peripheral forms of SpA can be diagnosed in patients with a predominant presentation of enthesitis in the presence of some other features that can include amongst others such as psoriasis, inflammatory bowel disease, or a family history of SpA. Enthesitis, especially when occurring at multiple sites, and in the absence of true synovitis recognized as joint swelling, is therefore a symptom that can be misinterpreted as diffuse pain. According to the Assessment of SpondyloArthritis International Society (ASAS) criteria, SpA may be classified as peripheral and axial disease, with the latter subdivided into non–radiographic axial SpA (nr-axSpa) and radiographic ax-SpA.^81^ The use of advanced MRI of the whole spine and sacroiliac joints has facilitated the diagnosis of SpA, as active inflammation recognised as bone marrow oedema can now be appreciated to occur in the absence of traditional radiographic findings such as sacroiliac joint changes or the presence of syndesmophytes.^10^ These classification criteria allow for earlier diagnosis and also diagnosis of atypical disease, especially in patients who did not clearly fit into a specific disease category, and has also allowed for a greater recognition of disease in women.

Patients with SpA may not specifically localize the pain to spinal locations, especially when costochondral and costovertebral junctions, as well as enthesopathic sites are involved. Enthesitis is clinically identified as local tenderness at the insertion of ligaments into bone at various locations such as the trochanteric regions, iliac crest regions, epicondyles of elbows, and classically Achilles tendon insertions, and may be confused with the body tenderness of FM.

In a study of 61 patients with inflammatory back pain, one-third fulfilled criteria for FM on tender point examination demonstrating overlap with enthesopathic sites of tenderness.^75^ Similar to FM, patients with SpA may experience fatigue, sleep disturbance, and depression.

The clinical overlap between FM and early forms of SpA may in fact be so great that nothing but a positive MRI could differentiate these 2 conditions. Again, even such a finding cannot with certainty solve the dilemma as to whether the patient has an overlap inflammatory disorder and FM or merely an early inflammatory disorder with no need for the additional diagnosis of FM. This distinction has both therapeutic and diagnostic importance.

The red flags for inflammatory spinal pain due to SpA include a gradual onset of pain especially in a person younger than 45 years, pain in the second half of the night and worse in the early morning, pain somewhat relieved by exercise, and stiffness in the mornings for longer than an hour. The location of axial pain may also provide a clue, as pain may change location over time, as for movement from low back and buttock regions, to cervical, midthoracic or anterior chest wall regions. However, many patients with FM may describe spinal symptoms compatible with inflammatory back pain. Associated psoriasis, uveitis, or bowel symptoms that could suggest an inflammatory bowel disorder should prompt consideration of SpA. In a study of patients diagnosed with FM in Israel, 10% met ASAS criteria for axial SpA, raising the question of either underdiagnosed SpA, or SpA with associated FM.^3^ Further investigation with appropriate imaging, including MRI of the spine and sacroiliac joints, as well as measurement of the human leucocyte antigen-B27 and CRP will serve to support a diagnosis of SpA.

## 5. Noninflammatory musculoskeletal conditions

### 5.1. Myofascial pain syndrome

Myofascial pain syndrome (MPS) is a regional muscular pain disorder, typified by latent and active trigger points which are discrete, focal, hyperirritable areas, usually within a taut band of skeletal muscle or in the muscles fascia. MPS may occur throughout the body, but with particular focus in spinal areas and is a main cause for referral to specialist pain clinics.^87^ Recent estimates indicate that over 44 million people in the United States alone have MPS. Similar to FM, the diagnosis of MPS relies solely on the physical examination as there are no laboratory or radiographic findings associated with either MPS or trigger points. The distinction between FM, believed to be mostly a central pain sensitization, and the peripheral nociceptive MPS could be challenging. Indeed, some authors suggested that peripheral nociception is an important pain generator in FM.^23,31^ This overlap has recently been demonstrated by Ge et al.^40^ who showed that the tender point sites in patients with FM are actually trigger points and therefore may function as peripheral pain generators.

The red flags to suggest a myofascial syndrome is pain that is more pronounced and localized, especially in the neck, shoulder, and low back regions, and the association of “trigger points.”^79^ Still, the concept of “trigger points,” including the purported differences between active and latent trigger points, remains contentious especially as trigger points are inconsistently assessed and the interrater reliability of assessment is of low quality.^5,70^ Manual therapists, however, advocate that this distinction should be made to direct treatments of trigger points with manual and stretching techniques.^79^

### 5.2. Hypermobility syndrome

Generalized joint laxity is a common finding in normal persons without physical complaints, with population prevalence rates reported to vary from 2 to over 50%.^77^ Joint laxity is most common in younger females, those of Asian, Middle Eastern, and African descent, and diminishes with age. Joint hypermobility may also occur in the setting of various connective tissue disorders including Marfan syndrome and Ehlers–Danlos syndrome (EDS), as well as chromosomal and genetic disorders such as Down syndrome and homocystinuria. When hypermobile joints are associated with chronic musculoskeletal complaints, as occurs for approximately 3% of hypermobile persons, the term hypermobility syndrome is used. Hypermobility syndrome may be diagnosed when generalized joint hypermobility is accompanied by pain in ≥4 joints over a period ≥3 months and in the absence of other conditions that cause chronic pain.^77^ Hypermobility syndrome can however be associated with CWP, and not only pain localized to joints, but the exact pathogenesis of pain is unclear with the speculation that it may be caused by the mechanical changes or generalized hyperalgesia.^20,78^

Hypermobile joints are also a component of the criteria for EDS, a heritable disorder of the connective tissue characterized by skin extensibility and tissue fragility. Ehlers–Danlos syndrome has a number of variants, with type 3 (hypermobility type) often identified synonymously with hypermobility syndrome. Different from the other 4 types of EDS, type 3 has no specific genotype and diagnosis is based entirely on clinical findings. There is however still debate as to whether hypermobility and EDS are distinct conditions, although in their seminal paper classifying EDS and quoted as the Villefranche classification, Beighton et al. stated in their concluding remarks “although this approach is valid and useful, it relies heavily on the identification and subjective interpretation of signs that are semiquantitative……the result is frequent diagnostic confusion. Another example is the frequent misdiagnosis of joint hypermobility as a type of EDS.”^13^

Similar to FM, there is an emerging recognition of a high prevalence of psychological distress, psychosocial factors, and psychiatric disorders in persons with benign joint hypermobility and EDS.^21,48,77,83^ The presence of any pain symptom in persons with EDS has been significantly associated with the presence of a psychiatric disorder.^48^ There has even been recent suggestion to recognize a new neurocognitive phenotype characterized by core symptoms of anxiety and collagen hyperlaxity.^17^ Recent study has also identified orthostatic intolerance in a cohort of 80 patients with EDS hypermobility types compared with controls, offering an explanation for the frequent report of fatigue in these patients.^25^ Therefore, at this time, identification of hypermobility may be a plausible explanation for the pain, but with possible overlap with FM.

## 6. Nonrheumatic medical conditions

### 6.1. Endocrine and metabolic disorders

Endocrine and metabolic conditions that can mimic FM include hypothyroidism, hyperparathyroidism, acromegaly, and vitamin D deficiency. All these conditions may present with ill-defined symptoms of body pain and fatigue, but with other clues unique to each condition that can lead to appropriate testing. Patients with hypothyroidism may have a family history of thyroid disease and have weight gain, whereas hyperparathyroidism may be associated with constipation. Of note, there is a frequent association of autoimmune thyroid (Hashimoto) disease with and without hypothyroidism and FM.^4^ Excessive growth hormone as a cause of acromegaly, although a rare condition, will be associated with increased size of hands and feet, coarsening of facial features as well as body pain and stiffness. For each of these conditions, specific laboratory testing is available, with most guidelines suggesting a screen for these conditions in the assessment of diffuse pain.

There is an association between CWP and hypovitaminosis D. Vitamin D deficiency manifests as osteomalacia with symptoms of muscle weakness and generalized bone pain.^93^ The myopathy associated with severe vitamin D deficiency, presenting as muscle weakness and a waddling gait, can also be associated with diffuse pain. Severe vitamin D deficiency is most commonly seen in those with limited sun exposure, especially living in the northern hemisphere and wearing clothing that covers most of the body, but may also be a consequence of disturbed phosphate metabolism.^29^ An association of CWP and vitamin D deficiency has recently been reported in a meta-analysis comprising almost 2000 patients with a cut-off value (8–10 ng/mL vitamin D) recommended to better define the population with and without CWP.^49^ However, there is a cautionary note. There is currently a trend in the developed world to focus excessively on laboratory measurements of vitamin D in generally healthy persons without an “at risk” predisposition such as malabsorption, with risk of creating an artificial pandemic of deficiency of this nutrient.^61^

### 6.2. Gastrointestinal diseases

Celiac disease or a spectrum of non–celiac gluten sensitivity, the latter currently hotly debated, have many somatic symptoms, including myalgia and arthralgia that mimic FM.^9^ In an Italian cohort of 468 patients suspected of having non–celiac gluten sensitivity, 31% reported joint or muscle pain resembling FM.^91^ In contrast, when 178 patients with FM were evaluated for celiac-type symptoms, patients with FM reported significantly more symptoms than controls, leading the authors to suggest that some patients with FM may be harbouring celiac disease or gluten sensitivity.^36^ Of the 104 patients with irritable bowel disease and FM, celiac disease was identified in 6.7%, suggesting that additional screening for this subgroup of patients may be indicated.^74^

### 6.3. Infectious diseases

Fibromyalgia may develop after an infectious illness, most commonly viral, but a search for an infectious aetiology is not routinely required. Infectious diseases such as Lyme disease, hepatitis C infection, and human immunodeficiency disease may have symptoms mimicking FM, but any testing in this regard should be dependent on a clinical suspicion of these infections.^50,52^ Most importantly, a screening of antibodies against *Borrelia burgdorferi* in patients with CWP without a history of medically proven Lyme disease is not recommended because the rate of FM in patients after culture confirmed Lyme disease is low.^99^ In fact, post-Lyme disease syndrome, characterized by fatigue, musculoskeletal pain, and cognitive complaints that persist for 6 months or longer after completion of antibiotic therapy, without persisting *Borrelia burgdorferi* infection, can be considered as a secondary FM.^7^

### 6.4. Malignancy

Although the presence of a malignancy becomes evident with time, in the very early stages, before diagnosis, FM may occasionally be considered, particularly in the setting of ill-defined pain, fatigue, and change in global health status. Identification of other constitutional symptoms such as fever, weight loss, or night sweats should prompt further investigation.

## 7. Neurological conditions

### 7.1. Neurological diseases

Neurological conditions with widespread body pain include multiple sclerosis (MS), Parkinson disease (PD), and peripheral neuropathies. The incidence and prevalence of FM seem to be slightly higher in MS compared with the general population.^62^ Furthermore, similarities between the 2 conditions include the neuropathic quality to the pain, which is present in a third of patients with FM, the fluctuating nature of symptoms, and presentation in younger women.^51,72^ Fatigue is a frequent accompaniment of both conditions. However, it is very unlikely that a diagnosis of MS would be made purely on a presentation with pain without any neurological symptoms in the history or physical signs at presentation.

### 7.2. Spinal stenosis and myelopathy

Although spinal stenosis is classically described as causing claudication symptoms especially when the stenosis affects the lumbar region, symptoms may be less clearly defined with some patients presenting with more vague and ill-defined body pain.^63^ This is especially true when the stenosis is located in the cervical region and symptoms may fluctuate over time and tend to move location similar to the pain complaint of FM. Causes of spinal stenosis may include degenerative disc disease, facet joint osteoarthritis, and congenital deformity of the spinal canal. Another cause of proliferative bony changes that can contribute to spinal stenosis is diffuse idiopathic skeletal hyperostosis.^59^ However, diffuse idiopathic skeletal hyperostosis as a unique diagnosis, although previously recognized as an incidental radiographic finding, can also be a cause of CWP in view of multiple locations of enthesopathy.^58^ Abnormality of the spinal canal can be identified by imaging studies such as MRI or computer tomography, whereas a simple radiograph of the thoracic spine can show bridging osteophytes across at least 3 vertebral spaces and also ossification of the anterior longitudinal ligament.^59^ It has to be taken into account, however, that abnormalities in spinal imaging correlate very poorly with symptoms, such that a diagnosis based on spinal MRI abnormalities has to be very critically evaluated in the context of the clinical picture.^18^

### 7.3. Myopathies and myositis

Although conditions affecting the muscles mostly present with muscle weakness, pain may also be present in a widespread distribution. Myopathies may be classified as congenital, metabolic, drug-induced myopathy, and inflammatory myopathy, with muscle weakness the key clinical abnormality. In new-onset myopathy, red flag symptoms that a patient may describe include difficulty climbing stairs, or standing from a seated position, even with normal muscle power on physical examination. Other history clues may point to a familial occurrence, changes in symptoms related to exercise or dietary intake of carbohydrates, or other systemic features present in a connective tissue disease such as skin rash in a heliotrope pattern on the face, “mechanics hands,” Raynaud phenomenon, skin tightening, or respiratory symptoms. Myositis as a paraneoplastic phenomenon must be remembered, especially for those with dermatomyositis. Macrophagic myofasciitis is a localized inflammation in a muscle that has been injected with a vaccine.^41^ Despite the local nature of the inflammation, the pain can be widespread and resemble CWP.^73^

Various inherited myopathies, such as late-onset Pompe disease, an autosomal inherited disorder with deficiency of acid maltase with only mild elevation of creatine kinase may masquerade as FM.^39^ In McArdle disease, where the enzyme myophosphorylase is missing, patients develop pain with exercise that may subside after 10 minutes of aerobic exercise (“second wind”), reminiscent of the relief that patients with FM may get with low-intensity training.^55^ Myoadenylate deaminase, another genetic enzyme deficiency, is considered by some authors to be a cause of myalgia, fatigability, and cramps,^85^ but others contend its pathogenicity.^44^ In myotonic dystrophy type 2, patients have varied types of pains, and pain can be the only symptom for years.^37,38^ Myotonic dystrophy type 2 has been reported to have been misdiagnosed as FM, with clues to the diagnosis in retrospect being proximal weakness of the lower limbs, the presence of myotonia and at times elevated creatine kinase.^8^ Neuromyotonia, often associated with autoantibodies to Caspr2, is characterized by muscle tenderness, continuous muscle activity (myokymia), cramps, and stiffness, and is not usually mistaken for FM. However, patients with FM could observe occasional muscle fasciculations and may present for diagnostic workup of neuromyotonia.

Finally, severe vitamin D deficiency may also present with myopathy, which is usually painless, but may have a component of pain, mostly due to mechanical factors related to weakness.^93^

## 8. Mental disorders

Chronic widespread pain is not a diagnostic criterion of any mental disorder. Pain is one of the diagnostic criteria of generalized anxiety disorder and somatoform pain disorder. The higher the level of care and patient selection, the higher (up to 80%) is the prevalence of mental disorders such as anxiety, depression, and posttraumatic stress disorder in patients with FM.^6^ However, not all patients with CWP/FM met the criteria of a mental disorder and vice versa.^27^ Therefore, mental disorders do not mimic CWP/FM but have a negative impact on their outcome. A screening of mental disorders in patients with CWP/FM is, therefore, recommended by some recent guidelines.^2,27,35^

## 9. Medication-induced pain conditions

Medications that can be associated with body pain (multisite myalgias and arthralgia) and can be confused with FM are listed in Table 4. These include statins, opioids, some chemotherapeutic agents, aromatase inhibitors, and bisphosphonates.^1,47,67^

**Table 4:**
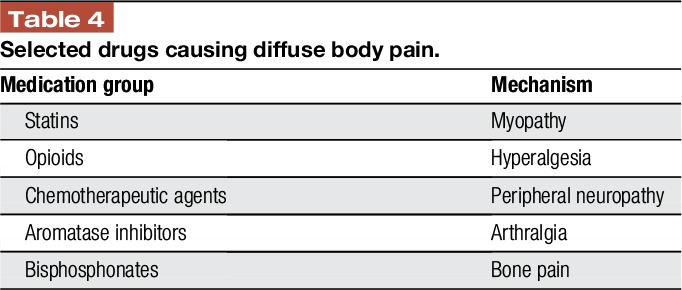
Selected drugs causing diffuse body pain.

Characteristically, the myopathy associated with statins or fenofibrate is painful, occurs early in the treatment phase, and is associated with an elevated creatinine kinase, although this measurement may be normal.^65^ Between 2% and 10% of patients receiving statins report myalgias, but the incidence is probably higher because the clinical trials of statins were not specifically designed to assess muscle-related adverse events.^12^ In case of moderate to severe muscle pain and/or weakness, discontinuation of the drug is recommended and symptoms are reversible when the drug is stopped, and should disappear within 2 months.^71,84^ This is in contrast to the rare statin-induced autoimmune myopathy that needs to be treated with immunosuppressants.^64^

Another class of drugs that may induce widespread body pain is opioids, with the induction of opioid-induced hyperalgesia.^46^ Although mostly recognized to occur in the setting of high-dose opioid treatment, hyperalgesia may occur early in opioid therapy and also with low doses of opioids. The pain may have a burning quality, can fluctuate during the day, and may not necessarily be associated with symptoms of withdrawal such as sweating or agitation. As the reported use of opioids by patients with FM in North America is over 30%, opioid-induced hyperalgesia may be an unrecognized factor perpetuating or aggravating symptoms of FM.^34^ It could be argued that as patients with FM have been reported to have decreased central μ-opioid receptor availability, either due to decreased concentration or downregulation of receptors, this dysregulation could explain hyperalgesia in these patients, and possibly a predisposition to the hyperalgesic effects of administered opioids.^45,80^ It should be noted, however, that true evidence supporting the existence of opioid-induced hyperalgesia as a common phenomenon is relatively limited.^28^

Chemotherapy-induced neuropathy, although mostly recognized as neuropathic symptoms in a glove and stocking distribution, can be a cause of a more generalized pain syndrome. Anecdotally, it has also been observed that some patients with a previous background of low-grade FM may develop a more severe exacerbation of diffuse pain with neuropathic qualities after chemotherapy (author's personal experience).

Aromatase inhibitors cause musculoskeletal pain in up to 50% of women treated for breast cancer.^16^ Although the symptom is mostly described as bone or joint pain, the generalized nature of the pain may be similar to that of FM in 20% of the cases.^54^ Similarly, bisphosphonates can cause bone, joint, and muscle pain that can occur either shortly after initiation or months or even years later, leading the Food and Drug Administration to issue an alert.^30^ The occurrence of body pain related to bisphosphonates is likely greatly underreported in view of the mechanism of postmarketing reporting of serious adverse events. Polymyositis and other myopathies have also been reported in postmarketing data and in the medical literature in association with proton pump inhibitor use.^22^ A red flag indicating a potential relationship between drug use and CWP might be the sudden onset of a multisite pain within 1 to 2 months after the initiation of a new drug therapy.

## 10. Fibromyalgia as a comorbid disease to other primary illnesses

There is increasing recognition that FM may occur in association with other chronic pain conditions, with most attention to date focussed towards the rheumatic diseases. Concomitant FM not only has impact on quality of life in the moment, but also adversely affects outcome, such as the success of total joint replacement in the setting of OA.^80^ Other pain conditions such as mechanical low back pain, visceral pain conditions, including chronic pelvic pain, chronic headaches, and temporomandibular joint pain may also be associated with a sensitization phenomenon, or spreading and amplification of the pain that is clinically recognized as FM.^101^ Failure to appreciate this broader spectrum of suffering will compromise clinical care.

### 10.1. Comorbid fibromyalgia in rheumatic diseases

Fibromyalgia may occur concomitantly with various medical conditions, but is most commonly recognized to coassociate with rheumatic conditions.^43^ It is reported that between 20% and 30% of patients with various inflammatory rheumatic diseases experience an associated diffuse pain syndrome. Using a self-reported questionnaire, a modification of the preliminary American College of Rheumatology (ACR) FM criteria, Wolfe et al.^94^ reported that criteria for FM were fulfilled for 17% with osteoarthritis, 21% with RA, and 37% with SLE. It has also been reported that those with an inflammatory rheumatic disease and concomitant FM report greater functional impairment, poorer quality of life, and more severe symptoms. Fibromyalgia was identified in 21.4% of patients with SpA, and associated with more enthesitis, higher disease activity, and more functional impairment.^14^

Although the primary medical condition carries major weight for treatment choices, failure to recognize the presence of concomitant FM may adversely affect outcome. Fibromyalgia may be erroneously interpreted as poor control of the underlying disease, with focus towards the primary disease rather than attention to FM. Therefore, a treatment approach that addresses FM symptoms rather than using disease-modifying interventions or biological treatment is required. In contrast, increased activity of an underlying inflammatory rheumatic disease which might be due to loss of effect of current treatments or poor adherence to treatments may be misinterpreted as FM.

### 10.2. Comorbid fibromyalgia in neurological diseases

Chronic widespread pain may also be a concomitant symptom of various neurological diseases. In the early stages of PD, patients may perceive a stiffness which may be interpreted as pain.^86^ Although studies to date report conflicting results for pain in PD, with some reporting pain similar to controls, 2 recent studies report an increased prevalence of pain.^26,66^ It is possible that up to a two-thirds of patients with PD report some type of pain.^86,92^ Similar to FM, symptoms of PD may develop gradually over time, patients may be fatigued and have mood disturbance, and laboratory testing is normal. Parkinson disease usually occurs later in life and with a male predominance. However, when other signs and symptoms of disease, in particular, the motor signs are still subtle, presence of pain may be misleading and result in a delayed diagnosis especially in a woman. A patient with arm pain has been reported, in whom PD was diagnosed 2 years later with the development of tremor in her right leg.^42^

Neuropathic pain, although characteristically mostly presenting as distally accentuated limb pain with burning pain of the hands and feet, can also be widespread in some patients. In a study of 32 patients with hereditary neuropathy with liability to pressure palsy, almost half reported musculoskeletal pain, with a third meeting the FM criteria.^100^ Another area of debate is the comorbid association of FM with small fiber neuropathy. Although reductions in epidermal innervation have been shown in FM,^89^ for some authors the 2 conditions should not be confounded,^88^ but for some authors small fiber neuropathy represents the main pathophysiological mechanism of FM.^19^

## 11. Conclusions

Although the most likely reason for a complaint of CWP is FM, this pain complaint can be a harbinger of a number of conditions other than FM, prompting consideration of a differential diagnosis. The clinical encounter, which encompasses a thorough medical and psychosocial history and a full clinical examination, is critical in the assessment of CWP. With laboratory and various technical diagnostics mostly of lesser importance than a sound clinical evaluation, the assessment of CWP exemplifies the essence of practicing the “art of medicine.” Launching an extensive “fishing expedition” with an abundance of unnecessary and often costly investigations is poor medical practice and detrimental to care. Therefore, the diagnoses of FM can mostly be established in the primary care setting, based on a history of a typical cluster of symptoms, a physical examination without findings indicating a somatic disease and normal basic laboratory testing without the need for specialist referral. When there is reasonable suspicion of some other somatic disease that presents with CWP, referral to a specialist with a specific question may be needed.

In this review, we have described various conditions that may mimic FM and have highlighted features that can help to differentiate them from FM. By categorizing these conditions as systemic inflammatory rheumatic diseases, noninflammatory musculoskeletal conditions, nonrheumatic medical conditions, neurological disorders, mental health disorders, and medication-related pain conditions, health care professionals will be more alert to other diagnostic possibilities in the setting of CWP. Furthermore, the recognition that FM may occur as a comorbid condition with some other underlying disease, as supported by the recent modification of the ACR 2016 criteria for FM, opens the door to consideration of this diagnosis in a wider spectrum of patients and will importantly affect health outcomes if neglected.

## Disclosures

The authors have no conflicts of interest to declare.
